# Hydrolyzed Yeast Supplementation to Newly Weaned Piglets: Growth Performance, Gut Health, and Microbial Fermentation

**DOI:** 10.3390/ani12030350

**Published:** 2022-01-31

**Authors:** Waewaree Boontiam, Chaiyaphum Bunchasak, Yoo Yong Kim, Sumetee Kitipongpysan, Jinsu Hong

**Affiliations:** 1 Division of Animal Science, Faculty of Agriculture, Khon Kaen University, Khon Kaen 40002, Thailand; 2 Department of Animal Science, Faculty of Agriculture, Kasetsart University, Bangkok 10900, Thailand; agrchb@ku.ac.th; 3 School of Agricultural Biotechnology, Research Institute of Agriculture and Life Sciences, Seoul National University, Gangnam-ru, Seoul 135-754, Korea; yooykim@snu.ac.kr; 4 Division of Agriculture, Faculty of Agriculture and Life Science, Chandrakasem Rajabhat University, Bangkok 10900, Thailand; sumetee.k@chandra.ac.th; 5 Department of Animal Science, South Dakota State University, Brookings, SD 57007, USA; jinsu.hong@sdstate.edu

**Keywords:** early-weaned practice, swine, productive performance, immunity, inflammation, brewer yeast

## Abstract

**Simple Summary:**

Early-weaning in piglets has negative effects on growth performance and gut health, which may cause economic losses in the swine production worldwide. Therefore, this study aimed to examine the effects of a highly digestible protein ingredient from hydrolyzed yeast (*Saccharomyces cerevisiae*) on growth performance, nutrient digestibility, gut health, and microbial fermentation in early-weaned piglets. Our study found that supplementing hydrolyzed yeast increased growth performance, crude protein digestibility, villus height, villus height-to-crypt ratio, and immunity and decreased inflammation and fecal pathogen count compared with those fed a diet with no addition of hydrolyzed yeast. These research outcomes indicate that supplementation of hydrolyzed yeast has the potential to enhance the growth performance and gut health of early-weaned piglets.

**Abstract:**

Hydrolyzed yeast (HY)-derived protein from *Saccharomyces cerevisiae* has a high digestible protein content and nucleotides and is a sweetener immunostimulatory substance. This could be used in nursery diets to minimize diarrhea and improve the growth rate and gut health of early-weaned piglets. This research was conducted with the objective of examining the effect of the inclusion level of HY as a potential protein ingredient for early-weaned piglets. A total of 72 crossbred weaned piglets [(Landrace × Large White) × Duroc] were assigned to three dietary treatments in six replicates with four pigs per pen. Dietary treatments were: (i) control (CON), piglets weaned at 18 days; (ii) CON diet with 5% HY inclusion (HY5); and (iii) CON diet with 10% HY inclusion (HY10) in a corn–soybean meal-based basal diet. Increasing HY levels positively improved body weight, average daily gain, and average daily feed intake (linear effect, *p* < 0.05). Furthermore, there was a linear increase in N-retention, albumin, jejunal villus height, villus height-to-crypt depth ratio, immunoglobulin A, acetate and propionate production, and *Lactobacillus* spp. count proportional to the dose of the HY-supplemented diet (*p* < 0.05). It also observed a decrease in diarrheal rate, jejunal crypt depth, blood urea nitrogen, pro-inflammatory cytokines, branched amino acids, and *E. coli* corresponding to the HY-supplemented levels (*p* < 0.05). However, the changes in the apparent total tract digestibility (dry matter, crude ash, and crude fat), blood glucose, butyrate, and *Salmonella* spp. were unaffected by the dietary HY level. Therefore, the supplementation of HY in the diet for early-weaned pigs not only supported the growth rate and immune function but also activated the beneficial bacterial growth of the early-weaned piglets.

## 1. Introduction

Early weaning of piglets is an advantageous practice as it increases sow productivity and prevents pathogenic transmission from their sows. However, the young piglets are more susceptible to various stressors, that can subsequently increase disease susceptibility and lead to economic losses. [1,2]. To overcome this escalating problem, it is important to get young piglets to consume more feed and gain body weight quickly as it affects days to market. The typical nutritional approach is to formulate diets by utilizing various feed ingredients with highly digestible and palatable ingredients containing functional amino acids, as well as supplemented with additives [3]. A recent report has demonstrated that a highly digestible protein ingredient shows effects against antioxidant and anti-inflammation mechanisms, while modulation function is beneficial for gut microbiome [4]. However, the shortage of plant-based protein resources has been the major limiting factor in driving sustainable development in the swine industry, thus, contributing to the search of an alternative feed ingredient.

Though hydrolyzed yeast (HY) derived from brewer’s yeast cells (*Saccharomyces cerevisiae*) activates the cell’s rupture through specific enzymatic hydrolysis, it has been in the swine industry for many decades. It contains high amounts of digestible crude protein (41.3% of dry basis) and all essential amino acids (2.71% of lysine), including glutamic acid (4.16%) [5]. It also represents a unique composition of nucleotides, microbial enzymes, mannan oligosaccharides (MOS), and β-glucans, which is not fully understood [5,6]. These components are important for RNA transcription, cell proliferation, intestinal homeostasis, and immunity that may positively affect growth performance of young animals [7,8]. According to Moehn et al. [9], the incorporation of 9% yeast-derived protein led to a similar apparent and standardized ileal digestibility of amino acids as spray-dried plasma protein in young piglets. The addition of 4% dried brewer’s yeast in creep and nursery diets also promoted the growth performance of nursing and weaning piglets [10]. Furthermore, the 4% HY-supplemented diet not only promoted the health of the animal but also activated antioxidant enzyme function and *Lactobacillus* growth and reduced inflammation [5]. However, the incorporation of a novel HY ingredient for early-weaned piglets is not fully understood. We hypothesized that the use of HY as a high digestible protein ingredient might promote animal health and feed utilization of 18-day weaned piglets. To test this hypothesis, we investigated the effect of various levels of novel HY on growth performance, apparent nutrient digestibility, gut health, and microbial fermentation in early-weaned piglets.

## 2. Materials and Methods

The procedures of the trial (authorization No. IACUC-KKU4/64) were reviewed and approved by the Institutional Animal Care and Use Committee of Khon Kaen University (Khon Kaen, Thailand).

### 2.1. Hydrolyzed Yeast Component

The HY component contains crude protein (41.84%), ether extract (2.3%), calcium (0.06%), phosphorus (0.71%), gross energy (4682 kcal/kg), β-glucan (22.43%), and MOS (15.6%).

### 2.2. Animal Care, Housing, and Treatment

A total of 72 mixed crossbred piglets ([Landrace × Large White] × Duroc; 5.71 ± 0.22 kg initial body weight) with the same number of males and females, weaned at 18 days, were obtained from the swine research unit of Khon Kaen University (Khon Kaen, Thailand) in two batches of 36 piglets. The healthy piglets with no symptoms of disease were assigned to three dietary treatments with six pens of four pigs (two gilts and two barrows) in each pen, containing three pens per batch in a randomized complete block design. Treatments were (i) control (CON; 18 ± 2-day weaning (*n* = 24) without HY supplementation); (ii) CON + 5% of HY-supplemented diet (HY5); and (iii) CON + 10% of HY-supplemented diet (HY10). A corn–soybean meal (SBM)-based diet was prepared weekly to ensure feed quality in mash form using a horizontal feed mixer with a maximum capacity of 150 kg. The diet with no addition of HY was prepared prior to the HY-supplemented diet to avoid feed contamination among treatments. The latter was mixed at increasing levels of HY. The piglets were fed a mash diet according to a two-phase feeding program: Phase I, 1 to 14 days post-weaning; and Phase II, 15 to 28 days post-weaning (Table 1). The experimental diets were formulated to meet or exceed the predicted requirements for weaning pigs [11], having similar metabolizable energy, Ca, and standardized ileal amino acid content. All pigs had available access to feed and water during the study.

Each pen (1.6 × 1.6 m, with a stocking density of 0.64 m^2^ each) had slatted concreted floors and was equipped with a nipple drinker, polyvinyl dry feeder, and heating lamp. Unused rice straw as bedding was provided twice daily at 06:00 and 19:00 during a 14-day period to minimize temperature stress under partially controlled conditions, which conformed to the European regulations (EU Directive 2010/63/EC for animal experiment). The housing environment was controlled using mechanical ventilation to maintain a desirable temperature of 31 ± 1 °C during the 1st week, and this was gradually decreased to 29 ± 1 °C, which was maintained throughout the entire experiment. There was no occurrence of atrophic rhinitis, transmissible gastroenteritis, Aujeszky’s disease, Salmonellosis, or porcine reproductive and respiratory syndrome during the previous three years; therefore, vaccination was not administered during the study.

### 2.3. Growth Performance

On days 14 and 28 post-weaning, each pig’s body weight (BW), the amount of feed supplied, and feed disappearance were recorded on a pen basis at the end of each feeding phase. These values were used to calculate the average daily gain (ADG), average daily feed intake (ADFI), and feed efficiency (gain to feed ratio, G:F). Growth performance criteria were summarized in each feeding program and for the overall period.

### 2.4. Diarrheal Occurrence

All piglets were observed daily for health parameters by two technicians. The visual assessment of fecal consistency was carried out on a pen basis and divided into 4 categories: 0 = hard feces (normal), 1 = brown and soft stool feces (normal), 2 = yellow or bloody soft formed sticky feces (diarrhea), and 3 = watery diarrhea (severe diarrhea). The diarrheal occurrence was reported as the number of diarrheal piglets/(total number of piglets x diarrhea day) × 100. Mortality was represented as the percentage of dead pigs (number of dead pig/number of initial pigs in the group).

### 2.5. Nutrient Digestibility

Twelve crossbred barrows (11.23 ± 0.43 kg of initial BW) were assigned to three treatments (four replicates) with a completely randomized design in individual metabolic pen (0.85 m × 1.15 m × 0.68 m), placing a tray underneath a metabolic cage for 5 days of feed adaptation to the diet and to acclimate the experiment condition. Each pen was equipped with a cup drinker near the stainless-steel feeder and concrete grate flooring, kept at a temperature of 31 ± 1 °C. Pigs were fed diets mixed with chromic oxide and ferric oxide (3 g/100 g of feed) as indigestible initial and terminal markers, respectively. Fecal sampling was performed for 5 days. Fecal samples were collected daily from the mesh bottom of each crate at 07:00 and 19:00, and sealed in plastic bags at −20 °C. These representative samples of feces and completed feed (500 g per pen) were dried to constant weight at 60 °C for 72 h; then, they were ground in a centrifugal mill to have a particle size below 0.85 mm for further analyses. The homogenous samples were detected in triplicate for crude ash (method no. 942.15) and dry matter (method no. 930.15) using a forced air drying oven at 135 °C for 2 h; crude protein (method no. 984.13; N × 6.25) was measured using the Kjeldahl method, and ether extract (method no. 920.39) using the Soxhlet apparatus as per AOAC guidelines [12]. Nutrient digestibility was examined and calculated using the formula of Adeola [13]. Urine was collected daily for 5 days using plastic buckets containing 50 mL of 10% H_2_SO_4_, covered with a glass-wool filter to eliminate unwanted matters. Each sample was subsequently diluted with 4000 mL tap water to obtain a homogenous mix prior to storage in 50 mL conical tubes for determination of nitrogen retention.

### 2.6. Intestinal Morphology

On day 28, pigs were deprived of feed for 12 h. Six pigs (three gilts and three barrows; *n* = 18) having a normal BW close to the average pen BW were removed for slaughter at a local slaughterhouse (Kasetsombun, Chaiyaphum, Thailand). Approximately 5 cm pieces of duodenum (approximately 70 cm caudal from the pyloric region of stomach) and jejunum (5 cm between stomach sphincter and ileo-cecal junction) were collected and flushed with phosphate-buffered saline and then fixed in 10% (*v*/*v*) saline solution before being delivered to the laboratory (Betagro Science Center, Pathumthani, Thailand). Intestinal tissues were cut into pieces of 5 µm thickness from paraffin blocks using a rotary microtome prior to drying in an incubator at 37 °C. The slides were subsequently deparaffinized with xylene and rehydrated with ethanol for staining with hematoxylin and eosin. Photomicrographs were detected with 40× magnification using a light microscope equipped with stereological software (Olympus Corporation, Tokyo, Japan). Six well-oriented villi with the entire villi and crypt covered in the cross-section and presented in the central lacteal were chosen to measure villous height (VH: from the villous tip to the villous–crypt junction). Crypt depth (CD) was measured from the villous–crypt junction to the villous bottom; hence, the ratio of VH to CD was calculated.

### 2.7. Blood Collection, Metabolic Profiles, and Immunity

On day 14, blood samples were collected from 18 pigs (one pig per pen in an equal sex), whereas the collection on day 28 was performed before euthanization. A 12 mL blood sample was obtained from individual pigs through jugular venipuncture into two sets of serum tubes coated with micronized silica particles (True Biomedical, Pathumthani, Thailand; 6 mL/tube). The collected samples were harvested for serum using a centrifugal force at 1872× *g* for 15 min at 4 °C and the supernatant was transferred to microcentrifuge tubes prior to being frozen (−80 °C) pending assays for metabolic profile and immune response assessment.

Serum samples were thawed at room temperature for 60 min. The serum concentrations of glucose, albumin, and blood urea nitrogen (BUN) were quantified using a colorimetric method, following the manufacturers’ instructions (Cambridge Biomedical, Cambridge, UK). Briefly, 20 μL of serum was adjusted the volume to 50 μL with glucose assay buffer (Glucose Assay Kit, Abcam, Waltham, MA, USA) or albumin assay buffer (Albumin Assay Kit, Abcam, Waltham, MA, USA) and mixed for 60 s to ensure homogeneity. These representative samples were used to quantify glucose and albumin at the absorbance of 570 and 620 nm, respectively. For BUN determination, serum was diluted in a 10-fold dilution with distilled water. Then, 50 μL of the diluted sample was placed on each microplate, and we added 75 μL of acid solution and incubated them for 30 min before determination, performed at an absorbance of 450 nm. All blood measurements of glucose, albumin, and BUN concentration were detected by spectrophotometer.

Serum immunoglobulin A (IgA) concentration was quantified using an Enzyme-Linked Immunosorbent Assay (ELISA) porcine kit (E101-102; Bethyl Laboratories, Inc., Montgomery, LA, USA). Briefly, 10 μL of diluted serum with phosphate-buffered saline with a final dilution of 1:100,000 was pipetted into each microplate, followed by the addition of 100 μL of biotinylated porcine IgA, enzyme-conjugated antibody, and color reagent, and incubated for 60 min at room temperature. The assays of interleukin-1β (IL1) and interleukin-6 (IL6) were performed using the ELISA kit (R&D System, Minneapolis, MN, USA). One hundred microliters of standard and samples were pipetted into a 96-well plate with a plate sealer, followed by incubation at room temperature for 30 min to aspirate liquid. For the IL1 assay, the samples were coated with 90 μL of chromogen TMB substrate solution and diluted hydrochloric acid as the detection reagents after incubation. For IL6 assays, the commercial kit had a detection range from 125 to 8000 pg/mL. After incubation, an aliquot of 100 μL of detection antibody and streptavidin–HRP were added as the working dilution, followed by 50 μL of 2 N H_2_SO_4_ as the stop solution. Tumor necrosis factor alpha (TNFα) was quantified using the ELISA porcine immunoassay kit (R&D System, Minneapolis, MN, USA). Fifty microliters of sample were added to each plate coated with biotinylated antibody reagent. Detection was performed using 3,3′, 5,5′ tetramethybenzidine, and a stop solution of 2 mol/L H_2_SO_4_. The absorbance for all measurements was detected at 450 nm using a spectrofluorometer in triplicate to avoid variation, with a total of 48 samples (six samples per treatment).

### 2.8. Microbial Fermentation

To examine microbial fermentation in pig feces, fresh fecal samples (10 g) were collected directly from each pig’s rectum after slaughtering and divided into two parts. Samples (six samples per treatment, *n* = 18) were pooled on a pen basis. The bacterial enumeration was detected using commercial agars—MacConkey, Salmonella–Shigella, and *Lactobacillus* medium II agars (Difco Laboratories, Detroit, MI, USA)—to determine *Escherichia coli*, *Salmonella* spp., and *Lactobacillus* spp., respectively. Each agar was prepared by suspending the dehydrated agar in 1000 mL of distilled water, boiling, and autoclaving at 121 °C for 15 min. One gram of the composite fecal sample was diluted with 9 mL of 10 g/kg buffered peptone broth (Becton Dickinson, Franklin Lakes, NJ, USA) and homogenized properly using vortex mixing for 15 min. One milliliter of diluted feces was overlaid on the specific agar (15 mL per plate) and incubated at 37 °C under aerobic conditions after solidification of the medium for 24 to 48 h to allow bacterial growth. The visible spotted colonies were counted immediately based on their morphology and color. The number of each microbial was expressed as the logarithm of colony-forming units per gram of feces, for a total of 48 samples (duplicate). Another fecal sample was snap-frozen in liquid nitrogen and immediately used to quantify short-chain fatty acids (SCFA). Briefly, feces were diluted with deionized water (1:1; w/v), mixed, and centrifuged at 1872× *g* for 10 min. After filtration, the supernatant fraction of 0.1 mL was added to 1.0 mL of 2-methyvaleric acid (catalog # SHBL3457) as an internal standard before injection for gas chromatography (CP-3380 GC, Varian, Inc., Walnut Creek, CA, USA). The initial temperature of the column was 170 °C and the injection temperature was held at 240 °C (run time 25 min). Hydrogen was used as a carrier gas at a flow rate of 5.0 mL/min, and the split rate was 70 mL/min. One microliter of sample was made at a split ratio of 1:10. The concentrations of SCFA were quantified from the peak area and presented as µmol per gram of feces.

### 2.9. Statistical Analysis

All the data were analyzed using the general linear model procedure of SAS (version 9.4, SAS Institute Inc., Carry, NC, USA) in a randomized complete block design. Each pen (*n* = 18) was an experimental unit for the growth performance assays, whereas each pig was an experimental unit for nutrient digestibility, intestinal morphology, blood components, immunity, and microbial fermentation. The orthogonal polynomial contrasts were tested for linear and quadratic effects in response to the dosage of HY. The statistical significance for a tendency was detected at *p* > 0.05 to *p* < 0.10. Results were reported as the least square mean and pooled standard error of the mean.

## 3. Results

### 3.1. Growth Performance and Diarrhea Occurrence

The growth performance and diarrhea occurrence of weaning pigs throughout the study are summarized in Table 2. The piglets’ BW increased linearly with increasing levels of HY on days 14 (*p* = 0.005) and 28 (*p* = 0.048), which subsequently increased ADG in a linear manner during Phase I (*p* = 0.001) and the overall period (*p* = 0.034). Linear and quadratic effects on ADFI (*p* = 0.005 and *p* = 0.033, respectively) were also observed during Phase II, including G:F ratio during Phase I (*p* = 0.001). In addition, a linear reduction in the piglets suffering from diarrhea was observed throughout the study (*p* = 0.028, *p* = 0.001, *p* = 0.001, respectively), which was in response to the supplemental level of HY. The quadratic effect of diarrhea occurrence was also detected in Phase II (*p* = 0.046) as a response to the HY dosage.

### 3.2. Nutrient Digestibility

The results of the experimental treatments on the apparent total tract digestibility of nutrients in weaning pigs are summarized in Table 3. The pigs fed the HY-supplemented diet showed a linear increase in crude protein digestibility (*p* = 0.032) and N retention (*p* = 0.015), whereas lower fecal N (*p* = 0.032) and urinary N (*p* = 0.031) excretions in response to the HY level were observed. However, the HY did not affect the digestibility of dry matter, crude ash, and ether extract.

### 3.3. Intestinal Morphology

The results of the experimental treatments on the intestinal morphology of the weaning pigs are summarized in Table 4. The piglets fed the HY-supplemented diet showed a linear decrease in crypt depth (*p* = 0.081) and increased VH:CD ratio (*p* = 0.001) in the duodenum in a dosage-dependent manner on HY addition. A positive change regarding the inclusion level of HY in the diet was linearly detected for jejunal villus height (*p* = 0.004), crypt depth (*p* = 0.001), and VH:CD (*p* = 0.001).

### 3.4. Metabolic Profiles

The results of the experimental treatments on the metabolic profiles in weaning pigs are summarized in Table 5. There was a tendency for improved albumin (*p* = 0.002) and decreased BUN (*p* = 0.035) concentrations on day 28 in a linear response to the HY level. However, glucose concentration remained unaffected throughout the study.

### 3.5. Immunoglobulins and Pro-Inflammatory Cytokines

The results of the experimental treatments on immunoglobulins and pro-inflammatory cytokine secretion in weaning pigs are summarized in Table 6. The HY supplementation was more effective in the reduction of all pro-inflammatory cytokine criteria on day 14 in a linear response (*p* < 0.05). The IL1 concentration also increased significantly in both a linear and quadratic manner during both periods (*p* < 0.05). Furthermore, significant reductions in IL1 (linear and quadratic effects; *p* < 0.05) and TNFα concentrations (linear effect; *p* = 0.012) as well as increased IgA concentration (linear effect; *p* = 0.003) on day 28 were influenced by the levels of supplemented HY.

### 3.6. Fecal Microbial Fermentation

The results of the experimental treatments on the fecal VFA content on day 28 are presented in Table 7. Increasing levels of HY in the diet led to a linear increase in acetic acid (*p* = 0.003), propionic acid (*p* = 0.009) concentrations, and SCFA:BCFA (*p* = 0.001) with lowered BCFA (*p* = 0.015). However, no significant differences were detected for butyric and valeric acid concentrations in pigs’ feces. Indeed, the benefits of promoting *Lactobacillus* spp. and controlling the *E. coli* population were observed as a linear response to the increasing inclusion level of HY (Table 8; *p* = 0.008 and *p* = 0.025, respectively). However, *Salmonella* spp. was not influenced by HY-supplemented level.

## 4. Discussion

### 4.1. Growth Performance and Diarrheal Occurrence

Several previous studies have shown the positive effects of yeast cells and the extracts from *S. cerevisiae* on the growth performance of weaning pigs [14,15]. This is in line with the improvements of ADG, ADFI, and G:F ratio in early-weaning piglets fed HY-supplemented diet with increasing inclusion levels. The reason for the improved ADG and ADFI is linked to HY, which could potentially affect palatability and aid in the secretion of several enzymes for the better hydrolysis of nutrients to maximize the growth of weaning pigs [16]. This is in agreement with a previous study observing that a glutamine-enriched diet with HY treatment strongly influenced the palatability of the diet and stimulated appetite in early-weaned pigs via increased ADFI [17]. However, a quadratic response of ADFI based on the inclusion level of HY may contribute to the satisfaction of nutrient needs. Although the supplementation of HY could improve the growth rate in phase I, this was no longer observed in phase II. This suggests that the dietary HY addition depends not only on the yeast-derived protein level but also on the physical health of the individual pigs. However, the early-weaned pigs fed the diet without HY supplementation had lower feed intake. This suggests that the pigs have to balance their physiological status during the critical period of post-weaning. This seems to be reflected in the higher percentage of diarrheal occurrence from days 1 to 14. However, the decreased diarrheal rate in piglets receiving the HY diet might be associated with improved nutrient digestibility and morphological structure [18]. Therefore, changes in growth performance and diarrheal occurrences could be attributed to the adaptation in the piglets’ intestinal barrier function.

### 4.2. Nutrient Digestibility

The linear improvement of the apparent total tract digestibility of CP, nitrogen (N) excretion, and N retention in the HY-supplemented diet was possible due to the highly digestible protein source. It is easily digested in young piglets and thus can be provided as a superior protein source in the nursery diet [19,20]. An increase in CP digestibility would indicate the increased digestibility of amino acids that are further used in the porcine intestinal cells. This study formulated an isonitrogenous experimental diet, implying that less indigestible protein remained in the hindgut. This is in agreement with the lower concentration of BCFA. In addition, the composition of glutamine in hydrolyzed yeast has been proven to increase mucosal digestive enzyme activity and absorption function in animals [16]. These enzymes can further utilize the macronutrients in the diet more efficiently prior to absorption, which is in accordance with the longer jejunum villi found in the current trial. This is similar to the previous reports that the inclusion of 1% HY increased the amount of digestibility crude protein in pigs [20,21]. To support performance of early-weaned pigs, it requires over 5% of high-digestible HY for greater nutrient utilization.

### 4.3. Metabolic Profiles

Changes in metabolic profiles reflect metabolic disorders in animals. Glucose has been established as the primary trigger to examine metabolic changes and endocrine functions [22]. The lack of difference in both periods could imply that weaned pigs can cope with weaning stress without the alteration of endocrine induction. However, HY inclusion above 5% has been shown to increase the albumin concentration. This activation could circulate various substrates throughout the animal body [23]. Furthermore, the dietary inclusion of HY decreased BUN in early-weaning pigs. This is consistent with the finding of Zhang et al. [21] in relation to the improvements in CP digestibility and N-retention; however, contrasting results were presented by Sampath et al. [19], who noted differences in yeast strain, minimal dose, diet composition, and pig age. Therefore, the supplementation of HY in the nursery diet could be utilized more efficiently in order to optimize the nutrient balance.

### 4.4. Immunoglobulin and Pro-Inflammatory Cytokines

A higher IgA concentration is likely attributed to the activation of humoral immunity on the mucosal surface. It plays an essential role in preventing the epithelial mucosa from pathogen invasion and maintaining mucosal homeostasis [24]. Based on the unique structure of the yeast-derived β-glucan of Saccharomyces cerevisiae, which has a thick cell wall (~115 nm) and small particles, it induces its interaction between the host immune system [18]. This consequently enhances macrophage colonization and activates the antimicrobial activity of mononuclear cells and neutrophils [25]. Piglet age is considered as an important challenging factor as it caused weaning stress and suppression of immune function in this study. The HY might be a potential IgA immunostimulant during the critical period of early weaning, thus controlling bacterial invasion as confirmed by the lower shedding of *Escherichia coli* found in this study.

Early-weaned pigs fed a diet without HY supplementation are susceptible to pro-inflammatory cytokine secretions. It immediately induces monocyte and macrophage production, and contributes to intestinal disorders [17,26]. This is consistent with our findings regarding intestinal histomorphology and microbial counts. However, the lower expression of cytokines in piglets fed an HY diet could be related to the presence of several active compounds, for example, glutamine plays a role in cell proliferation, whereas β-glucans and their binding to specific cell receptors (e.g., Dectin-1 and roll-like receptors) activate antigen-presenting cells for pathogen prevention [18]. The MOS can also interfere with type 1 fimbria adhesion of enteric bacteria through binding itself to the bacteria [27]. This mechanism causes a reduction in toll-like receptor 4 in the Peyer’s patches of the intestinal mucosa and thus lowers the secretion of pro-inflammatory cytokines [28]. In this study, we observed reductions in IL1, IL6, and TNFα on day 14, inconsistent with previous finding [28], whereby significant changes in IL1 and IL6 secretion were not observed. The discrepancies in the findings may be attributed to the HY source and their composition, while the included level, experimental challenge, and weaning age of piglets could also be contributing factors [15,28,29].

### 4.5. Intestinal Morphology

Changes in intestinal mucosa can determine the gut health status of piglets. Newly weaned pigs are usually susceptible to many stressors [30]. These subsequently cause detrimental effects on growth and gut health. Our study observed the positive effects of HY-supplemented diet on intestinal morphology during weaning stress in newly weaned pigs. We observed an improvement in intestinal microvillus length that translates to an increase in the absorptive surface area of the enterocytes, thereby promoting growth performance. Furthermore, there were relatively high amounts of nucleotides and glutamine (3.5% and 4.88%, respectively) which can be used as building blocks to synthesize other compounds for cell proliferation [7,8]. The greater availability of this genomic material could support enterocyte growth and mucosal development in the duodenum of early-weaned piglets. There was a consequent increase in villus height of early-weaned piglets, consistent with a previous report [31] that observed the effect of yeast-derived nucleotides on maintaining intestinal morphology. Furthermore, piglets fed an HY-supplemented diet have the ability to utilize nutrients and energy more efficiently for growth rather than enterocyte proliferation to repair the epithelial cell damage caused by pathogen infection or inflammatory response. This was observed by the lower crypt depth values in the duodenum, which is consistent with previous findings [31]. However, Keimer et al. [20] did not observe any changes in crypt depth in nursery pigs. These variations might be the result of influence of lower dosage, different hydrolyzed yeast species, and piglet age. Improvements in VH and VH:CD ratio in pigs fed HY-supplemented diet provided an absorptive surface area for nutrients, a contributing factor to enhanced health status and growth performance of early-weaned pigs in this study. It could be implied that the inclusion of up to 10% HY could provide a sufficient amount of nutrients that is required for new tissue proliferation in early-weaned piglets.

### 4.6. Fecal Microbial Fermentation

Short-chain fatty acids (SCFAs) are indicative criteria to determine fiber-fermenting microbes in the large intestine of pigs. The effects of HY supplementation on health as observed in this study are possibly linked to the enrichment of MOS at 15.6%. The mechanism of MOS’ effect on gut health is attributed to increased oligosaccharide fermentation into SCFAs (acetate, propionate, and butyrate). Once the SCFAs are absorbed in the large intestine, they are further utilized as energy source for the growth of four major fibrolytic species, *Fibrobacter intestinalis*, *Pseudobutyrivibrio ruminis*, *Clostridium cellulolyticum*, and *Coprococus entacus* [32]. This might be in accordance with our findings on the production of lactic acid bacteria, *Lactobacillus* spp. Nevertheless, the content of butyrate in fecal samples was unchanged when pigs’ age increased [32,33], which is consistent with our findings. The search for alternatives to balance between fiber and protein fermentation is one of the approaches to minimize post-weaning diarrhea in nursery pigs. Although the phase I diet contained higher digestible proteins from soybean meal, fish meal, and skimmed powder than the phase II diet, it seems that the HY-supplemented diet caused a positive reduction in BCFA in weaned pigs. This implies that piglets have the ability to utilize digestible proteins from hydrolyzed yeast more efficiently when provided at an early stage of growth. The VFA:BCFA ratio represents the end-products of fiber digestion, between carbohydrate and protein fermentation, in response to the dietary treatment. This supports our hypothesis that the inclusion of HY increases the fecal VFA:BCFA ratio via activation of fiber microbial fermentation in the hindgut; thereby, suppressing pathogen growth and pro-inflammatory cytokine secretion, while improving the growth performance of early-weaned piglets.

It has been established that the microbial colonization in the gastrointestinal tract rapidly changes after birth, thus modulating microbial colonization and immune development in the gut [34]. *Lactobacillaceae* are the second microbial colonizers in the pig colon, and are classified as lactic acid bacteria, which are normally present during an animal’s life [35,36]. These bacteria can produce various inhibitory compounds, such as bacteriocins, lactic acid, organic acids, hydrogen peroxide, and diacetyl [37]. This consequently increases the acidic environment in the gut, inhibiting either pathogen growth or the competition for binding sites and nutrients among harmful bacteria. This is confirmed by our previous report that showed lower microbial shedding of *E. coli* and *Salmonella* spp. in weaning pigs fed HY [19,38]. This supports the criteria of acetate and propionate concentrations as the main energy sources for the growth of beneficial bacteria, and lower diarrheal incidence in this investigation. However, Hu et al. [5] did not observe any reduction in *E. coli* in weaned pigs fed a 4% yeast-derived protein ingredient. This implies that increasing the level of HY over 5% at an early age for piglets could result in the long-term improvement of gut health and growth performance during the vulnerable post-weaning period.

## 5. Conclusions

The dietary supplementation of HY makes nutrients available for young piglets. Inclusion of up to 10% HY in the diet was shown to not only improve the growth performance of early-weaned piglets but also exerted positive effects on intestinal structure and function.

## Figures and Tables

**Table 1:** Ingredient and nutrient values of the experimental diet used in a two-phase feeding program (%, as fed basis) ^1^.

Ingredient	Phase I (Days 1 to 14)			Phase II (Days 15 to 28)		
	CON	HY5	HY10	CON	HY5	HY10
Corn	49.68	46.31	46.59	50.28	46.00	41.61
Soybean meal (45.6%)	29.13	25.31	16.48	25.40	21.71	18.19
Broken rice	10	10	10	15	15	15
Fish meal (58%)	4.5	4.5	4.5	4	4	4
Skimmed milk powder	4	4	4	2	2	2
Rice bran oil	0.24	2.43	5.98	0.25	3.22	6.13
Hydrolyzed yeast	0	5	10	0	5	10
L-Lysine HCl (78%)	0.16	0.16	0.16	0.31	0.31	0.31
DL-methionine	0.08	0.08	0.08	0.14	0.14	0.14
Dicalcium phosphate	1.61	1.61	1.61	2.02	2.02	2.02
Dried salt	0.35	0.35	0.35	0.35	0.35	0.35
Vitamin-mineral premix ^1^	0.25	0.25	0.25	0.25	0.25	0.25
Calculated Values (%)						
Metabolizable energy (kcal/kg)	3300	3300	3300	3300	3300	3300
Crude protein	22.50	22.50	22.50	20.50	20.50	20.50
Calcium	0.80	0.80	0.80	0.80	0.80	0.80
Total phosphorus	0.66	0.66	0.66	0.66	0.66	0.66
SID Lys	1.43	1.43	1.43	1.38	1.38	1.38
SID Met + Cys	0.65	0.65	0.65	0.65	0.65	0.65
SID Tryptophan	0.25	0.25	0.25	0.23	0.23	0.23
SID Threonine	0.87	0.87	0.87	0.84	0.84	0.84
SID Lys/ME	0.10	0.10	0.10	0.10	0.10	0.10
Crude fat	2.99	2.85	2.77	2.93	2.76	2.58
Fiber	3.36	3.11	2.63	3.14	2.88	2.62
Analyzed Values (%)						
Crude protein	22.03	22.13	22.34	20.61	20.48	20.53
Crude fat	2.87	2.90	2.84	2.84	2.64	2.60
Ca	0.78	0.81	0.80	0.79	0.80	0.82
Phosphorus	0.70	0.69	0.69	0.66	0.66	0.66

^1^ Provided per kilogram of complete diet: vitamin A, 4,000,000 IU; vitamin D3, 600,000 IU; vitamin E, 8 g; vitamin K3, 0.4 g; vitamin B1, 0.3 g; vitamin B2, 1 g; vitamin B6, 0.5 g; vitamin B12, 4 mg; niacin, 4 g; choline chloride, 30 g; calcium pantothenate, 3 g; biotin, 10 mg; folic acid, 0.1 g; cobalt as cobalt sulfate, 0.2 g; copper as copper sulfate, 40 g; ferrous as ferrous sulfate, 36 g; manganese as manganese sulfate, 16 g; zinc as zinc sulfate, 20 g; iodine as potassium iodide, 0.2 g; selenium as sodium selenite, 0.02 g; and ethoxyquin, 10 g.

**Table 2:** Growth performance and diarrheal incidence of early weaned piglets fed HY-supplemented diet ^1^.

Criteria	Dietary Treatment			SEM	*p*-Value	
	CON	HY5	HY10		Linear ^2^	Quadratic ^3^
BW (kg)						
Day 0	5.74	5.69	5.71	0.216	0.928	0.914
Day 14	7.66	8.48	8.83	0.228	0.005	0.427
Day 28	13.94	15.17	15.38	0.453	0.048	0.381
ADG (g/day)						
Days 1 to 14	137.23	198.90	222.74	10.360	0.001	0.167
Days 15 to 28	448.63	478.25	468.33	34.566	0.695	0.651
Overall	292.93	338.57	345.54	15.165	0.034	0.322
ADFI (g/day)						
Days 1 to 14	297.04	311.00	313.33	9.360	0.245	0.622
Days 15 to 28	624.51	645.04	643.16	3.680	0.005	0.033
Overall	460.83	478.16	478.33	5.465	0.047	0.229
G:F ratio						
Days 1 to 14	0.460	0.646	0.711	0.036	0.001	0.204
Days 15 to 28	0.719	0.742	0.729	0.056	0.903	0.794
Overall	0.636	0.710	0.724	0.036	0.115	0.504
Diarrhea Rate (%)						
Days 1 to 14	9.24	6.53	4.67	1.256	0.028	0.788
Days 15 to 28	4.66	0.86	0.52	0.622	0.001	0.046
Overall	13.42	5.92	3.96	1.404	0.001	0.139

CON = control (18-day weaned piglet with unsupplemented HY); HY5 = CON + 5% hydrolyzed yeast; HY10 = CON + 10% hydrolyzed yeast. ^1^ Values show means of six replicates (pen) per treatment. ^2^ Linear effect of HY-supplemented level (*p* < 0.05). ^3^ Quadratic effect of HY-supplemented level (*p* < 0.05).

**Table 3:** Apparent total tract digestibility and nitrogen retention of weaning pigs fed HY-supplemented diet ^1^.

Criteria	Dietary Treatment			SEM	*p*-Value	
	CON	HY5	HY10		Linear ^2^	Quadratic
Dry matter	89.18	89.72	90.18	0.690	0.343	0.964
Crude protein	86.74	88.57	89.39	0.671	0.032	0.561
Crude ash	36.28	46.08	48.61	4.571	0.105	0.539
Ether extract	70.01	70.17	70.02	2.741	0.999	0.963
Nitrogen Retention (g)						
Fecal N	1.02	0.87	0.82	0.051	0.032	0.538
Urinary N	4.42	3.99	3.20	0.307	0.031	0.628
N retention ^1^	2.24	2.78	3.67	0.302	0.015	0.656

CON = control (18-day weaned piglet with unsupplemented HY); HY5 = CON + 5% hydrolyzed yeast; HY10 = CON + 10% hydrolyzed yeast. ^1^ Values show mean of four pigs per treatment (average BW 11.23 ± 0.43 kg); N retention = N intake (g)—Fecal N (g)—Urinary N (g). ^2^ Linear effect of HY-supplemented level (*p* < 0.05).

**Table 4:** Intestinal morphology of early weaned piglets fed HY-supplemented diet ^1^.

Criteria	Dietary Treatment			SEM	*p*-Value	
	CON	HY5	HY10		Linear ^2^	Quadratic
Villus Height (µm)						
Duodenum	364	397	412	20.4	0.128	0.719
Jejunum	359	408	430	13.3	0.004	0.419
Crypt depth (µm)						
Duodenum	246	229	218	10.5	0.081	0.865
Jejunum	252	230	202	6.8	0.001	0.726
Villus Height: Crypt Depth						
Duodenum	1.48	1.74	1.91	0.057	0.001	0.521
Jejunum	1.43	1.79	2.13	0.074	<0.001	0.879

CON = control (18-day weaned piglet with unsupplemented HY); HY5 = CON + 5% hydrolyzed yeast; HY10 = CON + 10% hydrolyzed yeast. ^1^ Mean values from six replicate pens with one pig per replicate pen (*n* = 18). ^2^ Linear effect of HY-supplemented level (*p* < 0.05).

**Table 5:** Metabolic profiles of early weaned piglets fed HY-supplemented diet ^1^.

Criteria	Dietary Treatment			SEM	*p*-Value	
	CON	HY5	HY10		Linear ^2^	Quadratic
Days 14						
Glucose (mg/dL)	67.36	63.87	54.44	8.522	0.309	0.781
Albumin (mg/dL)	3.22	3.56	3.68	0.825	0.702	0.918
BUN (mg/dL)	12.24	9.17	9.83	2.187	0.452	0.506
Days 28						
Glucose (mg/dL)	84.91	113.03	87.29	32.089	0.959	0.509
Albumin (mg/dL)	3.51	5.46	6.89	0.582	0.002	0.722
BUN (mg/dL)	16.74	10.64	11.37	1.562	0.035	0.105

CON = control (18-day weaned piglet with unsupplemented HY); HY5 = CON + 5% hydrolyzed yeast; HY10 = CON + 10% hydrolyzed yeast; BUN = blood urea nitrogen. ^1^ Mean values from six replicate pens with one pig per replicate pen (*n* = 18). ^2^ Linear effect of HY-supplemented level (*p* < 0.05).

**Table 6:** Immunoglobulin and pro-inflammatory cytokines of early weaned piglets fed HY-supplemented diet ^1^.

Criteria	Dietary Treatment			SEM	*p*-Value	
	CON	HY5	HY10		Linear ^2^	Quadratic ^3^
Days 14						
IgA (mg/mL)	0.42	0.72	0.82	0.113	0.032	0.491
IL1 (pg/mL)	53.23	27.67	30.41	5.138	0.011	0.048
IL6 (pg/mL)	117.82	85.74	70.38	9.530	0.013	0.555
TNFα (pg/mL)	12.43	8.11	6.06	1.721	0.028	0.602
Days 28						
IgA (mg/mL)	0.52	0.84	0.93	0.073	0.003	0.252
IL1 (pg/mL)	106.04	62.78	82.63	6.552	0.030	0.003
IL6 (pg/mL)	153.19	116.81	129.18	16.131	0.318	0.246
TNFα (pg/mL)	32.89	19.26	17.14	3.636	0.012	0.225

CON = control (18-day weaned piglet with unsupplemented HY); HY5 = CON + 5% hydrolyzed yeast; HY10 = CON + 10% hydrolyzed yeast; IgA = immunoglobulin A; IL1 = interleukine-1β; IL6 = interleukine-6; TNFα = tumor necrosis factor alpha. ^1^ Mean values from six replicate pens with one pig per replicate pen (*n* = 18). ^2^ Linear effect of HY-supplemented level (*p* < 0.05). ^3^ Quadratic effect of HY-supplemented level (*p* < 0.05).

**Table 7:** Fecal short chain fatty acids (μmol/g of feces) contents of early weaned piglets fed HY-supplemented diet ^1^.

Criteria	Dietary Treatment			SEM	*p*-Value	
	CON	HY5	HY10		Linear ^2^	Quadratic
Acetic	84.58	100.78	133.29	8.594	0.003	0.457
Propionic	28.46	36.41	40.94	2.720	0.009	0.618
Butyric	15.83	24.23	23.63	4.134	0.212	0.395
Valeric	7.78	7.11	7.41	0.581	0.659	0.514
BCFA	8.91	6.75	5.60	0.795	0.015	0.619
SCFA:BCFA	17.27	27.81	38.71	3.165	0.001	0.964

CON = control (18-day weaned piglet with unsupplemented HY); HY5 = CON + 5% hydrolyzed yeast; HY10 = CON + 10% hydrolyzed yeast; SCFA = Short chain fatty acid; BCFA = branched fatty acids. ^1^ Mean values from six replicate pens with one pig per replicate pen (*n* = 18). ^2^ Linear effect of HY-supplemented level (*p* < 0.05).

**Table 8:** Microbial counts (log cfu/g feces) of early weaned piglets fed HY-supplemented diet ^1^.

Criteria	Dietary Treatment			SEM	*p*-Value	
	CON	HY5	HY10		Linear ^2^	Quadratic
*Lactobacillus* spp.	5.74	8.46	9.68	0.837	0.008	0.484
*Escherichia coli*	9.28	5.93	6.12	0.846	0.025	0.119
*Salmonella* spp.	6.24	4.36	5.63	1.399	0.765	0.379

CON = control (18-day weaned piglet with unsupplemented HY); HY5 = CON + 5% hydrolyzed yeast; HY10 = CON + 10% hydrolyzed yeast. ^1^ Mean values from six replicate pens with one pig per replicate pen (*n* = 18). ^2^ Linear effect of HY-supplemented level (*p* < 0.05).

## Data Availability

The data showed in this research are available within the article.
